# Diabetes, Inflammation, Proinflammatory Cytokines, and Diabetic Nephropathy

**DOI:** 10.1100/tsw.2006.179

**Published:** 2006-08-09

**Authors:** Juan F. Navarro, Carmen Mora

**Affiliations:** ^3^ Spanish National Research Council, Madrid, Spain; ^2^ Canarian Institute for Biomedical Research, University Hospital Nuestra Señora de Candelaria, Santa Cruz de Tenerife, Spain; ^1^ Nephrology Service, University Hospital Nuestra Señora de Candelaria, Santa Cruz de Tenerife, Spain

**Keywords:** Diabetes, Diabetic nephropathy, Inflammation, Proinflammatory cytokines

## Abstract

Diabetes and its complications have become a public health problem. Diabetic nephropathy is the main cause of renal failure. In spite of our higher knowledge on this complication, the intimate mechanisms leading to the development and progression of renal injury are not yet fully known. Activated innate immunity and inflammation are relevant factors in the pathogenesis of diabetes. Moreover, inflammation, and more specifically proinflammatory cytokines and other molecules with a relevant role within the inflammatory process, may be critical factors in the development of microvascular diabetic complications, including nephropathy. This new pathogenic perspective may lead to important new therapeutic considerations and new therapeutic goals for the treatment of diabetic nephropathy.

## INTRODUCTION

Diabetes mellitus, specially type 2 diabetes, is a public health problem that has reached the range of epidemic due to the rapidly increasing rates of the disease throughout the world. More important, the prevalence of type 2 diabetes will continue to rise in the next 20 years, with the great concern that this disease is rising rapidly in children and adolescents worldwide[1].

From this perspective, it is easily understandable that target organ complications secondary to diabetes will be one of the most important medical concerns in the next decades. A clear example is diabetic nephropathy (DN), "a medical catastrophe of worldwide dimensions," which has become the single-most frequent cause of end-stage renal disease[2].

## DIABETES MELLITUS: FROM A METABOLIC DISORDER TO AN INFLAMMATORY CONDITION

Perspectives on type 2 diabetes have changed substantially in the last decade with the notion that chronic, low-grade inflammation and activation of the innate immune system are closely involved in the pathogenesis of this disease. This hypothesis was first proposed in 1997 and 1998, and suggested that long-term innate immune system activation, resulting in chronic inflammation, elicited disease instead of repair leading to the development of type 2 diabetes[3]. Several cross-sectional studies in the general population, as well as in nondiabetic subjects, in individuals with impaired glucose tolerance (IGT)/impaired fasting glucose (IFG) and in newly diagnosed or established type 2 diabetic patients have shown that acute-phase reactants and proinflammatory cytokines, such as C-reactive protein (CRP), sialic acid, tumor necrosis factor-α (TNF-α), and interleukin-6 (IL-6), are positively correlated with measures of insulin resistance[4,5,6,7,8,9]. Furthermore, diabetic patients exhibit elevated levels of these parameters compared with nondiabetic control subjects[7,8,10,11,12].

The Atherosclerosis Risk in Communities Study was the first work to show that inflammatory markers predict the development of type 2 diabetes[13,14]. Subsequent studies, including the U.S. Women's Health Study[15], the U.S. Insulin Resistance and Atherosclerosis Study[16], or the European Prospective Investigation into Cancer and Nutrition (EPIC)-Postdam Study[17], have confirmed that circulating inflammatory markers, acute-phase reactants, and proinflammatory cytokines are strongly associated with the risk of developing type 2 diabetes. In addition, the findings that anti-inflammatory agents decrease the acute-phase response and may reduce the risk of developing type 2 diabetes are of great interest. Yuan et al.[18] reported that insulin resistance in genetically obese *fa/fa* rats and *ob/ob* mice is reversed by salicylates via an IκB kinase β–dependent mechanism. In the West of Scotland Coronary Prevention Study[19], assignment to pravastatin therapy resulted in a 30% reduction in the risk of developing type 2 diabetes, which may be related to the drug's anti-inflammatory properties (inhibiting release of cytokines by up-regulating peroxisome proliferator-activated receptor (PPAR)-α and -γ and inhibiting the NF-κb pathway)[20,21]. Finally, several studies with the recently introduced thiazolidinediones have reported a significant improvement in insulin sensitivity[22] as well as a decrease in the risk of progression to diabetes in subjects with a known high risk for developing this disease[23]. In addition to their actions as PPAR-γ agonists, glitazones have anti-inflammatory effects with inhibition of cytokine production, macrophage activation, and reduction of the levels of inflammatory markers such as CRP[24,25,26].

The mechanisms by which chronic inflammation can evoke type 2 diabetes are not completely clear, but probably a conjunction of aspects has to be taken into account, including genetic, metabolic, and environmental factors. It has been postulated that in type 2 diabetes and IGT, prolonged lifestyle or environmental stimulants cause maladaptation to the normal physiological responses, causing disease instead of repair[6]. It is known that adipose tissue is able to synthesize and release the main proinflammatory cytokines — TNF-α, interleukin-1 (IL-1), and IL-6 — and that inflammatory markers are associated with body fat mass. Proinflammatory cytokines and acute-phase reactants are involved in multiple metabolic pathways relevant to insulin resistance, including insulin regulation, reactive oxygen species, lipoprotein lipase action, and adipocyte function[27]. There are clear examples that show the relevant role of different molecules such as PPAR-γ, free fatty acids (FFA), GLUT4 (the insulin-responsive glucose transporter), and the suppressors of the cytokine signaling (SOCS) in proinflammatory cytokine-induced insulin resistance. Proinflammatory cytokines are associated with a significant down-regulation of PPAR-γ expression[28]. FFA are known to produce insulin resistance dose dependently in skeletal muscle and liver[29]. In muscle, through mechanisms that involve intracellular accumulation of long-chain acyl-CoA and diacylglycerol, activation of protein kinase C (PKC), and decreased tyrosine phosphorylation of insulin receptor substrate (IRS)-1/2[30,31], FFA inhibit insulin-stimulated glucose uptake[32,33]. Concerning hepatic insulin resistance, FFA are associated with increased hepatic diacylglycerol content, increased activities of PKC-δ and inhibitor of κB-kinase (IKK), enhanced activation of nuclear factor-κB, and increased expression of the inflammatory cytokines TNF-α and IL-1β[34]. Recent experimental studies have demonstrated that TNF-α is also able to impair insulin stimulation of glucose uptake and GLUT4 translocation to the plasma membrane. This cytokine, through activation of IKK in a p38 mitogen-activated protein kinase–dependent manner, produces serine phosphorylation of insulin receptor (IR) and IRS-1, impairing its tyrosine phosphorylation by insulin and the corresponding activation of phosphatidylinositol 3-kinase and Akt[35]. Furthermore, *in vitro* studies have shown that even very low TNF-α concentrations resulted in repression of GLUT4 gene transcription and decreased GLUT4 mRNA stability[36,37]. Finally, proinflammatory cytokine-induced insulin resistance has been related to SOCS proteins. Eight SOCS family members have been identified as inhibitors of cytokine signaling, suggesting that they play a role in the negative feedback control of cytokine pathway[38,39,40]. Diverse proinflammatory cytokines, including TNF-α, IL-1, and IL-6, are able to induce one or more SOCS proteins in different tissues[38,39]. It has been demonstrated that SOCS-1 and SOCS-6 inhibit insulin-dependent IR-directed phosphorylation of IRS-1[40]. On the other hand, specific polymorphisms in the TNF-α and IL-6 genes are variously associated with insulin sensitivity or resistance[41,42]. Therefore, activated innate immunity and inflammation are relevant factors in the pathogenesis of diabetes, with convincing data that type 2 diabetes includes an inflammatory component[6,27].

## PROINFLAMMATORY CYTOKINES AND DIABETIC NEPHROPATHY

Our knowledge on DN concerning different aspects, such as risk factors, clinical course, factors that influence the severity as well as the progression of renal injury, and the possibilities of effective therapeutic approaches, have significantly improved in the last years. Both metabolic and hemodynamic factors are critical for the risk of developing DN. However, in spite of this improvement in our knowledge on DN, from a pathophysiological point of view, the intimate mechanisms leading from chronic hyperglycemia to the development of renal injury are complex and not yet fully known.

A number of experimental and clinical studies have demonstrated the significant role of various inflammatory molecules in the setting of DN, including acute phase reactants, inflammatory cytokines, adhesion molecules, and chemokines. Dalla Vestra et al.[43] showed that patients with type 2 diabetes and overt nephropathy exhibit elevated levels of diverse acute-phase markers of inflammation and proinflammatory cytokines, including CRP, serum amyloid A (SAA), and IL-6, which were higher in subjects with increased glomerular basement membrane (GBM) width, a crucial lesion of diabetic glomerulopathy. Furthermore, they observed a significant association of GBM thickening with fibrinogen and IL-6. Chow et al.[44] showed that db/db mice, a model of type 2 diabetes and DN, exhibited an increased expression of intracellular adhesion molecule (ICAM-1), which promotes inflammation in glomeruli and tubules by increasing leukocyte infiltration and adherence, along with a marked increase in macrophage infiltration. In a model of diabetes and hypertension, Kelly et al.[45] demonstrated that urinary albumin excretion was reduced and renal function was preserved in rats treated with ruboxistaurin, an inhibitor of protein-kinase C-β, in spite of hyperglycemia and elevated blood pressure. PKC has various isoforms that are activated in diabetes and signal a number of cellular responses, including activation and expression of inflammatory mediators, such as proinflammatory cytokines[46]. In another study, Banba et al.[47] showed that diabetic patients had elevated urinary levels of monocyte chemoattractant protein-1 (MCP-1), with a significant association between urinary levels of albumina and MCP-1. These findings suggest that MCP-1 contributes to the initiation and progression of DN.

Hasegawa et al.[48] suggested for the first time that proinflammatory cytokines could participate in the development of DN. In that study, macrophages incubated with GBM from diabetic rats produced significantly greater levels of IL-1 and TNF-α than macrophages incubated with membranes of normal nondiabetic rats. A number of experimental studies have demonstrated that diverse intrinsic renal cells, such as endothelial, mesangial, glomerular, and tubular epithelial cells, are able to synthesize proinflamamatory cytokines, molecules that have been associated with significant renal effects. IL-1 increases vascular endothelial permeability and has been involved in the proliferation of mesangial cells and matrix synthesis, as well as in the development of intraglomerular microcirculatory abnormalities related to prostaglandin production by mesangial cells[49,50,51]. Regarding IL-6, this cytokine affects extracellular matrix dynamics at both mesangial and podocyte levels, stimulates proliferation of mesangial cells, increases fibronectin expression, and enhances endothelial permeability[43,52]. Finally, most attention has been focused on the implications of TNF-α in the setting of DN in the last years.

### Tumor Necrosis Factor-α and Diabetic Nephropathy

The synthesis of human TNF-α starts with the elaboration of a prohormone, a 26-kD membrane associated form, which either serves as a precursor for the soluble molecule or binds without processing to the TNF-α receptors via cell-to-cell contacts[53]. This cytokine is synthesized primarily by monocytes/macrophages, although diverse studies have demonstrated that intrinsic renal cells, including glomerular, mesangial, endothelial, and tubular cells, are able to produce inflammatory cytokines that play a role in controlling growth, biosynthetic activities, and functions of cells[54,55,56,57]. Recent experimental investigations by our group have demonstrated that renal mRNA expression for TNF-α is significantly increased by approximately 2.5-fold in diabetic rats compared to normal rats[58].

A variety of bioactivities suggests that this cytokine may promote the development of diabetic microvascular complications. TNF-α has been implicated in the hemodynamic disbalance between vasodilatory and vasoconstrictive mediators, which may result in alterations of glomerular blood flow and glomerular filtration rate[59]. It has also been reported that this cytokine is cytotoxic to glomerular, mesangial, and epithelial cells, and may induce direct renal damage[60,61]. McCarthy et al.[62] demonstrated that TNF-α was able to promote the local generation of reactive oxygen species with a subsequent alteration of the barrier function of the glomerular capillary wall, resulting in enhanced albumin permeability, independently of hemodynamic factors or effects of recruited inflammatory cells. Experimental studies in animal models of DN have demonstrated that urinary albumin excretion significantly correlates with renal cortical mRNA levels and urinary TNF-α excretion. Moreover, increased urinary TNF-α excretion, as well as increased TNF-α levels in renal interstitial fluid, preceded the significant increase in albuminuria[58,63]. Exposure of tubular epithelial cells to TNF-α significantly increased the synthesis and secretion of lymphocyte and neutrophil chemoattractant factors[64], as well as the cell surface expression of ICAM-1[65,66], which has been implicated in the development of renal injury in diabetes[66]. Finally, TNF-α has stimulatory effects on sodium uptake by proximal tubule cells[67], contributing to sodium retention and renal hypertrophy, typical alterations during the early stage of DN[68].

In addition to the observations from experimental investigations, clinical studies have found a direct and significant association between serum TNF-α and urinary protein excretion in diabetic patients with normal renal function and microalbuminuria, as well as in subjects with overt nephropathy and renal insufficiency[10,69]. On the other hand, urinary TNF-α levels are also elevated in diabetic patients with increased urinary albumin excretion, and furthermore, there is a significant rise of urinary TNF-α excretion as DN progressed. Moreover, multivariate analysis shows a significant and independent relationship between urinary TNF-α and urinary albumin excretion. Interestingly, in these studies, a significant correlation between serum and urinary concentrations of TNF-α was not found, suggesting an intrarenal production of this cytokine[10].

## ANTI-INFLAMMATORY THERAPIES FOR DIABETIC NEPHROPATHY

Nowadays, available therapies for prevention or slow progression of DN are based on strict metabolic control and treatment of hypertension with inhibitors of the rennin-angiotensin system[70,71,72,73,74,75]. However, these strategies provide imperfect protection, being necessary innovative approaches with novel treatments[76]. The vision of DN as an inflammatory disease triggered by altered metabolic factors opens new and important therapeutic perspectives.

Different studies in experimental models of DN have shown that the administration of substances with anti-inflammatory properties, including cyclooxigenase-2 inhibitors, mycophenolate mophetil, or mizoribine, were able to reduce the expression of mediators of renal injury as well as prevent the development of glomerular and tubulointerstitial damage[77,78,79]. More importantly, diverse recent clinical studies have demonstrated that some of these strategies based on the modulation of inflammatory molecules may have important translational implications.

The final product of the rennin-angiotensin-aldosterone system, aldosterone, has been implicated as an important factor mediating renal disease[80,81]. Rossing et al.[82] have shown in a short-term, randomized, double-masked, placebo-controlled, cross-over trial that addition of a low dose of the aldosterone blocker, spironolactone, to the recommended maxium doses of angiotensin-converting enzyme inhibitors and/or angiotensin II type 1 receptor blockers may offer beneficial renoprotection as reflected by reductions in blood pressure and albuminuria in type 2 diabetic patients with nephropathy. Interestingly, an experimental work in type 2 diabetic rats showed that spironolactone treatment significantly inhibited urinary excretion of (MCP-1) as well as the renal expression of this factor[83]. These experimental findings have been recently confirmed in patients with type 2 diabetes complicated by DN[84].

Evidence over the last decade has implicated PKC as an important mediator of diabetes-induced vascular dysfunction[85]. This enzyme is composed of different isoforms, various of which are activated in diabetes, especially PKC-β. This molecule signals a number of cellular responses, including oxidative stress, expression and/or activation of inflammatory mediators, cellular proliferation, and tissue fibrosis[86]. A clinical trial designed to evaluate the effect of ruboxistaurin, a specific PKC-β inhibitor, has been recently published. In this pilot study, Tuttle et al.[87] showed that ruboxistaurin had favorable effects on urinary albumin excretion and renal function in type 2 diabetic patients with nephropathy, with the albuminuria-lowering effect occurring early and sustained long term.

Pentoxifylline (PTF) is a methylxanthine-derivate phosphodiesterase inhibitor widely used in the treatment of peripheral circulatory disorders based on its beneficial hemorheological activity. In addition, PTF possesses significant inmmunological and anti-inflammatory properties. It has been demonstrated that PTF inhibits the accumulation of TNF-α mRNA and the transcription of the TNF-α gene, suppressing the synthesis of this cytokine[88,89]. In different models of renal disease, including lupus nephritis[90], crescentic glomerulonephritis[91], mesangial proliferative glomerulonephritis[92], and remnant kidney model[93] — in all of them with inflammatory mediators such as TNF-α or ICAM-1 playing a significant role — PTF has shown beneficial effects preventing or attenuating renal injury. Regarding DN, recent studies by DiPetrillo and Gesak[94], as well as by our group (unpublished data), have reported that PTF administration was able to prevent the increased renal TNF-α expression, synthesis, and excretion during experimental diabetes. In addition, PTF therapy ameliorated renal sodium retention and renal hypertrophy, the initial pathological changes associated with DN.

In addition to these experimental observations, clinical results support the efficacy of PTF as a therapeutic agent for DN. It has been shown that PTF reduces urinary protein excretion in diabetic subjects, both with normal renal function[95,96] and renal insufficiency[69]. Furthermore, addition of PTF to blockers of the rennin-angiotensin system, both angiotensin-converting enzyme inhibitors[97] or angiotensin II type 1 receptor blockers[98], has been associated with a significant reduction of urinary albumin excretion. Importantly, diabetic patients with residual albuminuria after long-term treatment with ARB may obtain a beneficial additive antialbuminuric effect of PTF, which is significant and directly related to a reduction of urinary TNF-α excretion[98]. Finally, in a very recent study in patients with proteinuric primary glomerular diseases, PTF significantly reduced urinary protein excretion, along with an increase of serum albumin. This beneficial effect occurred in close association with a reduction of urinary MCP-1 excretion[99].

## CONCLUSIONS

Based on estimations by the World Health Organization, by the year 2025, over 300 million people worldwide will have diabetes[100]. About 25–40% of patients with type 1 diabetes and 30–40% of type 2 diabetic subjects will develop DN, which has now become the single-most common cause of end-stage renal disease in the Western world, with over half of all patients on renal replacement programs now having diabetes[101]. The pathogenic vision of diabetes mellitus has substantially changed in the last years. The knowledge we have today on the pathogenesis of DN indicates that diabetes-associated alteration in diverse metabolic and hemodynamic pathways activate adhesion molecules, chemokines, growth factors, and cytokines. Therefore, DN can be viewed as an inflammatory disease triggered by disordered metabolism[102]. Current treatment of the nephropathy complication of diabetes is suboptimal in halting the progression of this complex disease. The recent insights on the role of inflammatory pathways in the development of renal damage in diabetes provide strong rationale for anti-inflammatory approaches to be used in the therapy of DN. Modulation of these inflammatory processes is emerging as an innovative strategy that can be fully translated into clinical treatments in a near future.

## ACKNOWLEDGMENTS

The studies by the authors have been supported by Fundación Canaria de Investigación y Salud (FUNCIS), Sociedad Española de Nefrología (SEN), and Asociación Científica para la Investigación Nefrológica (ACINEF).
